# Flavopiridol Protects Bone Tissue by Attenuating RANKL Induced Osteoclast Formation

**DOI:** 10.3389/fphar.2018.00174

**Published:** 2018-05-03

**Authors:** Zi’ang Hu, Yilei Chen, Lijiang Song, Jasper H. N. Yik, Dominik R. Haudenschild, Shunwu Fan

**Affiliations:** ^1^Department of Orthopaedic Surgery, Sir Run Run Shaw Hospital, School of Medicine, Zhejiang University, Hangzhou, China; ^2^Lawrence J. Ellison Musculoskeletal Research Center, Department of Orthopaedic Surgery, University of California, Davis, Davis, CA, United States

**Keywords:** flavopiridol, CDK9, osteoclast, bone resorption, NF-κB

## Abstract

Bone resorption and homeostasis is carried out by osteoclasts, whose differentiation and activity are regulated by the RANK/RANKL axis. Our previous studies using a mouse model of joint injury show that joint trauma induces local inflammation followed by bone remodeling. The transcription factor cyclin-dependent kinase 9 (CDK9) is the major regulator of inflammation, as CDK9 inhibitor flavopiridol effectively suppress injury-induced inflammatory response. The objective of this study was to investigate the underlying mechanism through which flavopiridol regulates bone resorption. The effects of CDK9 inhibition, by the specific-inhibitor flavopiridol, on bone resorption were determined *in vivo* using two distinct and clinically relevant bone remodeling models. The first model involved titanium particle-induced acute osteolysis, and the second model was ovariectomy-induced chronic osteoporosis. The effects and mechanism of CDK9 inhibition on osteoclastogenesis were examined using *in vitro* culture of bone marrow macrophages (BMMs). Our results indicated that flavopiridol potently suppressed bone resorption in both *in vivo* bone-remodeling models. In addition, CDK9 inhibition suppressed *in vitro* osteoclastogenesis of BMM and reduced their expression of osteoclast-specific genes. Finally, we determined that flavopiridol suppressed RANKL signaling pathway via inhibition of p65 phosphorylation and nuclear translocation of NF-κB. Summary, CDK9 is a potential therapeutic target to prevent osteolysis and osteoporosis by flavopiridol treatment.

## Introduction

Bone tissue homeostasis is maintained by the dynamic balance between osteoblastic bone formation and osteoclastic bone resorption (Seeman and Delmas, 2006). This balance is vulnerable to influence by physical, endocrine and paracrine stimuli under various physiological and pathological conditions. In some circumstances like aging, osteoarthritis, and endocrine diseases, the equilibrium is disturbed and results in an imbalance between bone formation and resorption, leading to metabolic diseases such as osteopetrosis and osteoporosis (Eastell et al., 2016).

Recent studies have demonstrated that the resorption or remodeling of bone tissue mainly depends on the differentiation of osteoclasts (Boyce et al., 2009), which is regulated by two essential cytokines, namely macrophage colony-stimulating factor (M-CSF) and receptor activator of nuclear factor kappa B ligand (RANKL) (Abu-Amer, 2013; Sucur et al., 2014). Osteoclasts are multi-nucleated cells derived from hematopoietic stem cells of monocytic lineage. M-CSF plays a central role in directing hematopoietic stem cells into the monocyte/macrophage lineage, and also in up-regulating RANK expression, which is required for osteoclast differentiation. RANKL is a transmembrane protein that belongs to the tumor necrosis factor (TNF) superfamily that is closely associated with both acute and chronic inflammation (Boyce and Xing, 2008; Xing et al., 2012).

Inflammation is a biological response to stressful events that trigger the transcriptional activation of primary inflammatory response genes (Hargreaves et al., 2009; Zippo et al., 2009). In the absence of inflammatory stimuli, transcription of these genes has already been pre-initiated by RNA polymerase II, but its progress is soon blocked before transcription can enter the elongation stage (Yamaguchi et al., 2013). During a stress response, the transcription factor cyclin-dependent kinase 9 (CDK9) is rapidly recruited to the promoter to phosphorylate RNA polymerase II, which then escapes promoter-proximal pausing and proceed to synthesize full-length mRNAs (Hargreaves et al., 2009; Zippo et al., 2009; Chen et al., 2014). Hence, CDK9 represents a key target for suppressing inflammatory gene activation to effectively modulate the inflammatory response.

In our previous studies (Yik et al., 2014; Christiansen et al., 2015; Hu et al., 2016), we discovered that inflammatory stimuli, such as a knee joint injury, resulted in marked up-regulation of pro-inflammatory cytokines like IL-1β, IL-6, and TNF-α. This cytokine up-regulation is followed by a substantial subchondral bone remodeling that potentiates osteoarthritis. Importantly, we find that CDK9 inhibition by flavopiridol prevents the induction of pro-inflammatory cytokines, this demonstrates the anti-inflammatory effect of flavopiridol is due to its ability to suppress transactivation of inflammatory genes (Yik et al., 2014). Furthermore, numerous studies (Sucur et al., 2014; Cauley et al., 2016) indicate that osteoclastogenesis is induced under inflammatory condition. This suggests a possible connection between flavopiridol’s function in inflammatory gene expression, bone loss, and osteoclastogenesis.

CDK9 has long been recognized as an important factor in regulating NF-κB activity (Barboric et al., 2001). Given the well-known role of NF-κB in inflammation and in RANKL signaling in osteoclast formation, we investigated the roles of CDK9 in osteoclast function, using two *in vivo* mouse models of bone remodeling: (1) acute titanium particles-induced osteolysis, and (2) chronic estrogen deficiency-induced osteoporosis. We also determined the molecular mechanism of the bone protective effect of flavopiridol treatment, by investigating the effects of flavopiridol treatment on NF-κB/RANKL signaling and osteoclastogenesis of bone marrow macrophages (BMMs) *in vitro*.

## Materials and Methods

### Titanium Particles

Pure titanium (Ti) particles (1–3 μm diameter) were obtained from Johnson Mathey Chemicals (Ward Hill, MA, United States) and prepared by continuous washing in 100% ethanol for 48 h to remove endotoxins. The Ti-particles were then re-suspended in sterile PBS at a concentration of 300 mg/mL and stored at 4°C. The endotoxin level of particle suspension was undetectable as determined by a Limulus assay (Biowhittaker, Walkersville, MD, United States), according to the manufacturer’s instructions.

### Flavopiridol Injection

The small molecule CDK9 inhibitor Flavopiridol (Sigma-Aldrich, St. Louis, MO, United States) was dissolved in DMSO to a stock concentration of 5 mg/ml. Flavopiridol (7.5 mg/kg) diluted in sterile saline was administered locally (osteolysis) or intraperitoneally (ovariectomy) via a 30-gauge needle immediately after animal surgery. Thereafter, the drug was administered daily for up to 14 days for the calvarial osteolysis model, or three times weekly for up to 8 weeks for the ovariectomy model. Control untreated animals received saline injections only.

### Calvarial Osteolysis Model

Animal studies were conducted in accordance with the principles and procedures approved by the Animal Care Committee of Sir Run Run Shaw Hospital, Zhejiang University. The *in vivo* mouse calvaria experiments were then performed. Briefly, healthy 8-week old female C57BL/6 mice were randomly assigned into three groups (*n* = 6 each group): sham operated with PBS control (sham), Ti-particles with PBS (vehicle), and Ti-particles with flavopiridol (7.5 mg/kg). After anesthesia, a midline incision was made to the calvaria; the periosteum was then scratched and lifted to expose the middle suture of the calvaria. 30 mg of Ti-particles was then embedded, with vehicle or flavopiridol. The incision was closed sterilely. No mortality was observed during or after particle implantation. Mice were able to retain normal activity throughout the duration of the experiment. 50 μl of PBS or flavopiridol (7.5 mg/kg) was subcutaneously injected into the surgical implant site daily. Mice were sacrificed at 3-, 7-, and 14-day post-operation, and the calvaria samples were harvested thereafter. The degree of particle-induced osteolysis was assessed using high-resolution micro-computed tomography (μCT) and histological staining.

### Mouse Ovariectomy Model

12-week old C57BL/6 female mice were generally anesthetized and subjected to either a sham operation or bilateral ovariectomy (OVX). Mice were randomly divided into three groups (*n* = 6 each group): sham (sham operation with PBS injection), vehicle (OVX with PBS injection), and CDK9 inhibitor (OVX with 7.5 mg/kg flavopiridol injection). 100 μl of each solution was injected intraperitoneally, three times a week for 8 weeks. All mice were then sacrificed at the end. Mouse body weights were recorded weekly, and uteri were isolated and weighed at the end-point to confirm the effects of OVX. Right femurs were processed for μCT analyses.

### Micro-CT Scanning

After mice were sacrificed, the calvaria or right femurs were harvested and fixed in 4% formaldehyde in PBS (pH 7.4) for 24 h and then analyzed using high resolution μCT (Skyscan 1072; Skyscan, Aartselaar, Belgium) with following settings: X-ray voltage, 50 kV; electric current, 500 mA; rotation step, 0.7. To reduce metal artifacts, the implanted Ti-particles were removed by thorough washing with PBS before μCT scanning. Scans were performed at 9-nm intervals. After reconstruction, a square region of interest around the midline suture of the calvarium or trabecular bone at the distal femoral epiphysis was chosen for qualitative and quantitative analysis. The bone volume to tissue volume ratio (BV/TV), trabecular number (Tb.N), trabecular thickness (Tb.Th), and trabecular spacing (Tb.Sp) were measured using the resident reconstruction program (Skyscan).

### Histological and Histomorphometric Analysis

After μCT scanning, the calvaria samples were decalcified in a sonic decalcification machine (DeCa Dx-100, Pro-cure medical technology, HK) for 1 day, and then embedded in paraffin. Histological sections were prepared for hematoxylin and eosin (H&E), as well as TRAP staining. Six sections per group were then examined with a microscope (Fujifilm, Tokyo, Japan) using 4, 10, and 20X objectives.

### Mouse BMM Preparation and *in Vitro* Osteoclastogenesis

Primary bone marrow stromal cells (BMSCs) were isolated from bone marrow aspirates from hind legs of 6-week old, female C57BL/6 mice. Briefly, cells were harvested from the femoral and tibia bone marrow and cultured in complete medium: α-MEM (Genom, Biotech., Hangzhou, Zhejiang, China) supplemented with 10% FBS, 1% penicillin/streptomycin, and 30 ng/mL M-CSF [A kind gift from and produced by Dr. An Qin’s lab (Zhai et al., 2014)] in a 37°C, 5% CO_2_ incubator. Cells were cultured for several days and washed with PBS during medium changes every other day to remove non-adherent cells. At ∼90% confluence, cells were washed with PBS three times and then trypsinized for 30 min to harvest the adherent BMMs. BMMs were then plated in 96-well plates at a density of 8 × 10^3^ cells/well, in triplicates, and cultured for 24 h. Cells were then treated with various concentrations of flavopiridol (0, 50, 100, or 200 nM) plus M-CSF (30 ng/mL), and RANKL (50 ng/mL) [A kind gift from and produced by Dr. An Qin’s lab (Zhai et al., 2014)]; media was changed every other day. After 5 days, cells were fixed and stained for TRAP activity. The numbers of TRAP positive multi-nucleated osteoclasts and the percentage of area occupied by osteoclasts relative to total area were determined using Image-Pro Plus software (Media Cybernetics, Bethesda, MD, United States).

### Cell Viability Assay

The anti-proliferative effect of CDK9 inhibition on BMMs was assessed with a cell counting kit-8 (CCK-8, Sigma-Aldrich, St. Louis, MO, United States) according to the manufacturer’s instructions. BMMs were plated in 96-well plates at a density of 2 × 10^4^ cells/well, in triplicates, and cultured in 100 μL complete α-MEM medium with 30 ng/mL M-CSF in the presence of different concentrations of flavopiridol for 24-, 48-, and 72-h. After washing three times with PBS, 10 μL CCK-8 buffer was added to each well, and incubated for 2 h. The absorbance at 450 nm (630 nm as reference) was measured with a plate reader (ELX800, Bio-Tek, Winooski, VT, United States).

### Bone Resorption Pit Assay

Bone marrow macrophages were seeded at a density of 8 × 10^3^ cells/well containing bovine bone slice (5 mm × 5 mm) (A kind gift from and produced by Dr. An Qin’s lab) in a 96-well plate in triplicates. After 24 h, cells were treated with 50 ng/mL RANKL, 30 ng/mL M-CSF, and 0 or 200 nM flavopiridol for 5 days. Cells adhered to bone slices were then removed by mechanical agitation and sonication. Resorption pits were visualized under a scanning electron microscopy (SEM; FEI Instr., Hillsboro, OR, United States), and the bone resorption area (darker shades) was quantified using Image J software (National Institutes of Health, Bethesda, MD, United States).

### Quantitative Polymerase Chain Reaction (PCR) Analysis

For the determination of mRNA expression, 10 × 10^4^ BMMs were seeded in 24-well plate and cultured in complete α-MEM medium containing M-CSF (30 ng/mL) and RANKL (50 ng/mL). Cells were then treated with or without flavopiridol (200 nM) for various times, and harvested by scraping and transferred to Eppendorf tubes, followed by cell lysis, total RNA isolation, and reverse transcription to generate cDNA, using the RNeasy Minikit (Qiagen, Valencia, CA, United States) and HIFIScript cDNA synthesis kit (CWBio, Beijing, China) according to the manufacturer’s instructions. 4 μl of cDNA was used for quantitative real time-PCR (in a final volume of 20 μl) performed in triplicate in an ABI Prism 7500 system (Applied Biosystems, Foster City, CA, United States) with SsoFast EvaGreen supermix (Bio-Rad, Hercules, CA, United States) according to the manufacturer’s conditions. mRNAs of interest and the housekeeping genes were amplified with equal efficiencies, results were normalized to 18S rRNA and calculated as fold-change in mRNA expression relative to untreated control. Probes used to detect mouse cDNAs are as follow:

18S:
Forward, 5′-AGGGGAGAGCGGGTAAGAGA-3′,
Reverse, 5′-GGACAGGACTAGGCGGAACA-3′;
NFATc1:
Forward, 5′-TCCACCCACTTCTGACTTCC-3′,
Reverse, 5′-AAGGCAGAATCATGGACGAC-3′;
TRAP:
Forward, 5′-CCATTGTTAGCCACATACGG-3′,-
Reverse, 5′-CACTCAGCACATAGCCCACA-3′;
cathepsin K (CTSK):
Forward, 5′-TCCGCAATCCTTACCGAATA-3′,
Reverse, 5′-AACTTGAACACCCACATCCTG-3′;
CTR:
Forward, 5′-ACCATTATCCACGCCATCAC-3′,
Reverse, 5′-GAAGAAGTTGACCACCAGAGC-3′;
c-Fos:
Forward, 5′-GTTCGTGAAACACACCAGGC-3′,
Reverse, 5′-GGCCTTGACTCACATGCTCT-3′;
DC STAMP:
Forward, 5′-GCTGTATCGGCTCATCTCCT-3′,
Reverse, 5′-AAGGCAGAATCATGGACGAC-3′.

### Western Blotting Analysis

Bone marrow macrophages were seeded in 6-well plates at a density of 6 × 10^5^ cells/well. After reaching 90% confluency, cells were then stimulated with 50 ng/mL RANKL in the presence or absence of 200 nM flavopiridol in complete α-MEM for 0, 5, 10, 20, 30, or 60 min, immediately followed by washing with ice-cold PBS to stop the reaction. Total protein was extracted from cells using radio immune-precipitation assay (RIPA) lysis buffer (Sigma-Aldrich, St. Louis, MO, United States). Lysates were centrifuged at 12,000 ×*g* for 15 min, and supernatants were collected. The protein concentration was determined using BCA protein assay (Thermo Fisher Scientific). Proteins were resolved on 10% SDS-PAGE gels and transferred by electro-blotting to PVDF membranes (Bio-Rad, Hercules, CA, United States). Membranes were blocked in 5% skim milk in TBST [50 mM Tris (pH 7.6), 150 mM NaCl, 0.1% Tween 20] at room temperature for 1 h and then incubated with primary antibodies diluted in TBST overnight at 4°C. Membranes were washed and incubated with horseradish peroxidase-conjugated goat anti-mouse immunoglobulin G (Abcam, Cambridge, MA, United States), followed by detection using an electrochemical luminescence reagent (Millipore, Billerica, MA, United States). Protein bands were visualized using the LAS-4000 Science Imaging System (Fujifilm, Tokyo, Japan). Primary antibodies (Cell Signaling, Danvers, MA, United States) used were: p65 (rabbit anti-mouse, 1:1000 dilution); phospho-p65 (rabbit anti-mouse, 1:500 dilution); ERK (rabbit anti-mouse, 1:1000 dilution); p-ERK (rabbit anti-mouse, 1:250 dilution). The secondary antibody used was goat anti-rabbit IgG (H++L) HRP (Multisciences, Hangzhou, Zhejiang, China).

### Immunofluorescence

Bone marrow macrophages were seeded in 6-well plates at a density of 6 × 10^5^ cells/well with coverslips on the bottom. After reaching 90% confluency, cells were then stimulated with 50 ng/mL RANKL in the presence or absence of 200 nM flavopiridol in complete α-MEM for 1 h, washed three times with ice-cold PBS, and fixed with 4% paraformaldehyde for 15 min. After fixation, coverslips were removed and placed on glass slides separately. Samples were blocked with 5% skim milk for 15 min and incubated with primary antibodies diluted in blocking buffer at 4°C overnight. Samples were then washed and incubated with secondary antibody for 2 h and DAPI for 10 min. The samples were then washed and mounted with Prolong Gold Anti-Fade Reagent (Cell Signaling, Danvers, MA, United States), and fluorescence signal was detected by fluorescence microscopy (BX51TRF, Olympus, Tokyo, Japan). Primary antibodies were used as described above and detected with goat anti-rabbit IgG (H+L), FITC (Multisciences, Hangzhou, Zhejiang, China).

### Statistical Analysis

All *in vitro* experiments were performed in triplicates, all *in vivo* experiments have *n* = 6, and data were expressed as means ± SD. Results were analyzed using SPSS version 16.0 (SPSS, Chicago, IL, United States). When appropriate, two-tailed, unpaired Student’s *t*-test was used to make comparisons between two groups, and one-way ANOVA for comparisons between multiple samples. *P* < 0.05 indicated a significant difference between groups.

## Results

### Flavopiridol Treatment Reduces Both Acute Osteolysis and Chronic Estrogen Deficiency-Induced Osteoporosis

**FIGURE 1:**
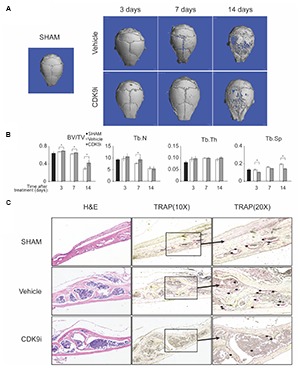
Flavopiridol treatment effectively prevents bone resorption in an acute titanium particle-induced osteolysis model. **(A)** Mice were injected with titanium particles and treated with vehicle or CDK9 inhibitor flavopiridol (CDK9i). Representative μCT scans of the calvarias at different times are shown. **(B)** The bone volume/tissue volume (BV/TV), trabecular number (Tb.N), trabecular thickness (Tb.Th), and trabecular separation (Tb.Sp) derived from μCT scans are shown. Histograms represent the mean ± standard deviation (*n* = 6 per group) (^∗^*P* < 0.05). **(C)** Histological assessment of mouse calvarial osteolysis by H&E and TRAP staining of 14 days’ samples. Arrows indicated TRAP-positive osteoclasts.

In this study, we evaluated the effects of flavopiridol treatment using two animal models with bone loss induced by Ti-particles or ovariectomy. In the Ti-particles model, osteolysis is acutely induced within 14 days. Loss of bone density at the calvaria was apparent in the vehicle treated animal group by day 7, and at day 14 there was extensive bone loss of >50%, when compared to the sham control (**Figures 1A,B**). The loss of bone was confirmed by the increase of trabecular spacing in the vehicle treated group (**Figure 1B**). In contrast, flavopiridol treatment significantly prevented bone loss at all time points tested when compared to vehicle treatment (**Figure 1B**). flavopiridol treatment also prevented the increase in trabecular spacing due to Ti-particles (**Figure 1B**). Trabecular number and thickness were not significantly different between the vehicle and CDK9 inhibition groups (**Figure 1B**).

The bone protective effect of flavopiridol treatment was further confirmed by histological analysis of the calvaria samples (**Figure 1C**). TRAP staining showed that multinucleated osteoclasts were significantly increased in the vehicle-treated group, but their numbers were reduced to a level similar to the sham control in the CDK9i group (**Figure 1C**). These results indicate that flavopiridol treatment prevents osteolysis in the calvaria by reducing the number of osteoclasts.

**FIGURE 2:**
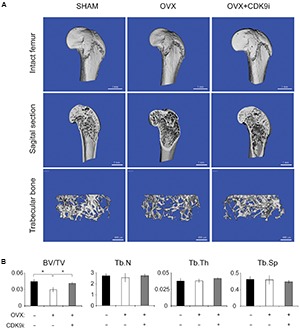
Flavopiridol treatment effectively prevents bone resorption in a chronic overiactomy-induced osteoporosis model. Mice were subjected to overiactomy and treated with vehicle or CDK9 inhibitor (CDK9i) flavopiridol. **(A)** The proximal femurs were scanned with μCT at 8 weeks and representative images in different treatment groups were shown. **(B)** The bone volume/tissue volume (BV/TV), trabecular number (Tb.N), trabecular thickness (Tb.Th), and trabecular separation (Tb.Sp) derived from μCT scans were shown. Histograms represent the mean ± standard deviation (*n* = 6 per group) (^∗^*P* < 0.05, paired *t*-test).

In the ovariectomy-induced osteoporosis model, systemic bone loss is induced by chronic estrogen deficiency and this allows us to investigate the bone protective effects of flavopiridol treatment in the long-term. Extensive trabecular bone loss in the proximal femoral head was observed by μCT in the vehicle-treated ovariectomy group when compared to the sham control (**Figures 2A,B**). However, flavopiridol treatment restored the trabecular bone density to a level similar to the sham group (**Figure 2A**). Similar trend was observed in trabecular number and thickness (**Figure 2B**). These results indicate that flavopiridol treatment is also effective in long-term bone protection.

### Non-cytotoxic Levels of Flavopiridol Inhibit Osteoclastogenesis *in Vitro*

Our data suggest that a potential mechanism of flavopiridol treatment in preventing bone resorption is due to a reduction in osteoclast numbers (**Figure 1C**). Other studies have reported anti-proliferative effects of flavopiridol at concentrations at or above 300 nM (Aktug et al., 2016; DiPippo et al., 2016; Shafer and Grant, 2016), which is the targeted plasma concentration for anti-cancer clinical trials (Senderowicz, 1999). However, studies have also suggested that the anti-proliferative effect of flavopiridol is likely due to indiscriminate inhibition of other CDKs that directly regulate cell-cylce, since only 3 nM of flavopiridol, or 100-fold less than the anti-proliferative concentration, is sufficient to inhibit CDK9’s activity (Chao et al., 2000). In light of these contradicting studies, we determined the cytotoxic profiles of flavopiridol in osteoclast precursor BMM in order to distinguish whether flavopiridol’s effects on osteoclastogenesis is due to non-specific pan-CDKs or specific CDK9 inhibition. BMM cultures were incubated with different concentrations of flavopiridol for 24, 48, and 72 h, and cell viability was measured and compared to that of untreated controls (Supplementary Figure 1). There was no significant cytotoxic effect at doses at or below 200 nM, and hence flavopiridol was used at 200 nM for the remaining studies.

**FIGURE 3:**
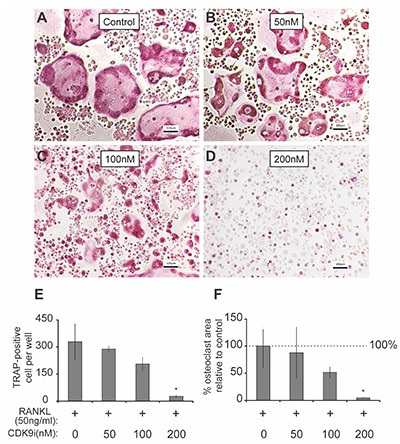
Flavopiridol treatment prevents osteoclastogenesis *in vitro*. **(A–D)** BMMs were treated with M-CSF (30 ng/mL), RANKL (50 ng/mL) and the indicated concentrations of flavopiridol for 5 days. Cells were stained for TRAP. The numbers **(E)** of TRAP-positive multinuclear cells and the surface areas they occupied **(F)** relative to control were determined as described in section “Materials and Methods.” Histograms represent the mean ± standard deviation (*n* = 3, ^∗^*P* < 0.05, one-way ANOVA). Results indicated that flavopiridol inhibits osteoclastogenesis in a dose-dependent manner.

We next determined the effects of flavopiridol on RANKL-induced osteoclast differentiation in BMMs. As shown in **Figures 3A–D**, flavopiridol significantly inhibited the formation of TRAP-positive multinucleated osteoclasts in a dose-dependent manner. The TRAP-positive cells decreased from ∼327/well (0 nM flavopiridol) to ∼27/well (200 nM flavopiridol) (**Figure 3E**), and the osteoclast-occupied area dropped from 100% (0 nM flavopiridol) to 4.68% (200 nM flavopiridol) (**Figure 3F**). Importantly, flavopiridol at 200 nM or below did not affect cell proliferation, as the total cell numbers (undifferentiated BMMs + osteoclasts) determined as cell density in culture plates were similar in all experimental groups. Collectively, these data show that 200 nM flavopiridol effectively inhibited osteoclastogenesis without arresting BMM cell cycle.

### Flavopiridol Treatment Suppresses Osteoclast-Specific Gene Expression *in Vitro*

**FIGURE 4:**
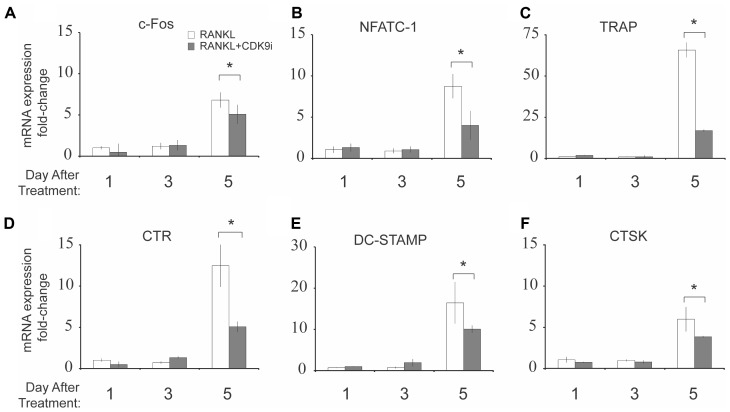
Flavopiridol treatment suppresses RANKL-induced osteoclast-specific gene expression *in vitro*. **(A–F)** Expression of osteoclast-specific genes in BMMs treated with M-CSF (30 ng/mL), and RANKL (50 ng/mL), and flavopiridol (200 nM) for 1, 3, or 5 days. Gene expression was analyzed by real-time PCR. Fold-changes in RNA expression for individual genes relative to untreated day 1 controls were normalized to the expression of 18S ribosomal RNA. Histograms represent the mean ± standard deviation (*n* = 3, ^∗^*P* < 0.05).

We next examined the effect of flavopiridol treatment on RANKL-induced mRNA expression of osteoclast-specific genes. BMMs were stimulated with RANKL in the presence or absence of 200 nM flavopiridol, and the mRNA expression of osteoclast-specific genes were then determined by quantitative PCR. As expected, RANKL treatment induced the expression of all osteoclast-specific genes by day 5 (**Figures 4A–F**). However, CDK9 inhibition significantly suppressed the RANKL-induced expression of all osteoclast-specific genes. In addition, housekeeping genes (e.g., 18S, GAPDH, ACTB) were not affected by flavopiridol treatment, indicating flavopiridol treatment ion specifically targeted RANKL-induced genes.

### Flavopiridol Treatment Prevents Osteoclastic Bone Resorption *in Vitro*

**FIGURE 5:**
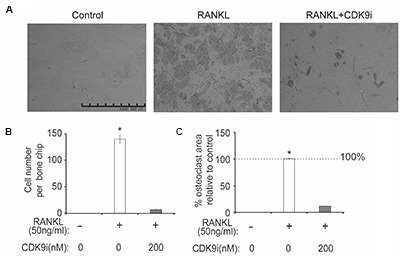
Flavopiridol treatment impairs osteoclastic bone resorption *in vitro*. BMMs were seeded onto bone slices and treated with the same conditions as described in **Figure 4** for 7 days. **(A)** Representative scanning electron microscopy (SEM) images of bone resorption pits were shown (scale bar = 500 uM). **(B)** Resorption pit areas were measured using Image J and **(C)** the percentage area of osteoclast relative to control was then calculated. Histograms represent the mean ± standard deviation (*n* = 3, ^∗^*P* < 0.05).

We next tested the effects of flavopiridol treatment on osteoclast function using *in vitro* bone pit formation assay. Bovine bone slices were cultured with BMMs and stimulated with RANKL, with or without 200 nM flavopiridol, and the formation of bone pits were examined by electron microscopy. As expected, osteoclast numbers, and areas of bone resorption (darker shades, **Figure 5A**) were significantly increased after RANKL stimulation. However, flavopiridol treatment almost completely abolished the effects of RANKL (**Figures 5A–C**). Together, these data show that CDK9 inhibition suppresses osteoclast-mediated bone resorption *in vitro*.

### Flavopiridol Treatment Suppresses RANKL-Signaling Pathway via NF-κB Phosphorylation and Nuclear Translocation

**FIGURE 6:**
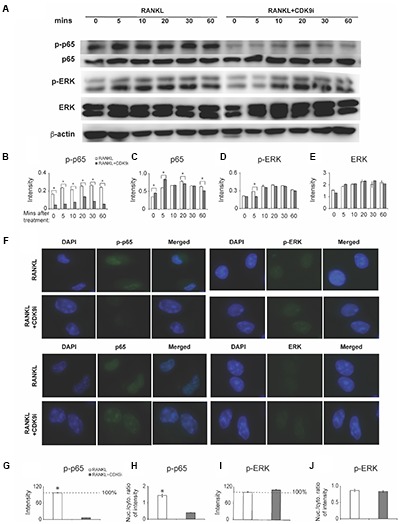
Flavopiridol treatment prevents osteoclastogenesis by targeting RANKL-induced NF-κB pathway. **(A)** BMMs were treated with 50 ng/mL RANKL in the presence or absence of 200 nM flavopiridol for 0-, 5-, 10-, 20-, 30-, and 60-min. The expression of total and phosphorylated p65, ERK, and β-actin was determined by Western blot. Images of bands were grouped together. **(B–E)** The intensity of each protein band was quantified and normalized to β-actin using Image J (*n* = 3, ^∗^*P* < 0.05). **(F)** BMMs were treated with CDK9 inhibitor (200 nM), M-CSF (30 ng/mL), and RANKL (50 ng/mL) for 1 h and total and phosphorylated p65 and ERK was detected by immunofluorescence imaging. Nuclear positions were marked by DAPI. **(G–J)** The fluorescence intensity of p65 and ERK in different samples was quantified with Image J. The signal of RANKL only samples was set to 100%. The nucleus VS cytoplasm signal intensity ratio of p-P65 and p-ERK was calculated to indicate the nucleus translocation (results were the mean ± standard deviation, ^∗^*P* < 0.05).

To elucidate the mechanisms by which flavopiridol treatment affects RANKL-induced osteoclastogenesis, we investigated key signaling pathways, including the NF-κB and MAPK signaling pathways. A time course study was performed on RANKL-treated BMMs, with or without 200 nM flavopiridol. The phosphorylation status of the key signaling proteins in the RANKL pathway, p65 (a component of NF-κB) and extracellular signal-regulated kinase (ERK), was detected by Western blotting. As shown in **Figure 6A**, RANKL treatment stimulated phosphorylation of both p65 and ERK. In contrast, CDK9 inhibition significantly attenuated phosphorylation of p65 (**Figures 6A–C**). However, flavopiridol treatment generally didn’t affect phosphorylation of ERK in the current study (**Figures 6A,D,E**). These data suggest that CDK9 inhibition suppresses NF-κB signaling pathways in BMMs.

We next measured nuclear translocation of p65 and ERK by immunofluorescence staining. The results showed that RANKL stimulation induced p65 phosphorylation and nuclear translocation (**Figures 6F–H**), which was abolished by flavopiridol treatment (**Figure 6F**), confirming the Western blot data above. Taken together, these data suggest that flavopiridol treatment blocks RANKL signaling pathway by targeting p65 and NF-κB. Meanwhile, no obvious suppression of the MAPK signaling pathway was found by CDK9 regulation in immunofluorescence staining (**Figures 6F,I,J**).

## Discussion

In this study, we determined that CDK9 is important in osteoclast differentiation and bone resorption function. The bone protective effect of CDK9 inhibition by flavopiridol treatment was demonstrated *in vivo* in two mouse models to examine conditions that lead to acute (Ti-particles) and chronic (estrogen-deficiency) bone loss.

Bone remodeling is a characteristic in many disease progressions, for example, during osteoarthritis pathogenesis. In a previous study using a mouse model of knee injury, injury-induced inflammation caused a rapid loss of tibia epiphysis trabecular bone mass (up to 30–40%) within 7 days of injury. This was followed by a gradual but only partial recovery (to ∼80%) of the original bone tissue by day 30 post-injury (Christiansen et al., 2015), indicating a bone-remodeling event occurs after a knee injury. The new bone formed from the remodeling was thinner and fenestrated due to a decrease in mineral density, which is associated with a reduction in the elastic moduli and bone strength (Mahjoub et al., 2012). In this case, preventing the bone loss/remodeling from happening in the first place is perhaps more beneficial than promoting the subsequent regaining of weaker bone tissue. Indeed, this study demonstrated that treatment with the CDK9 inhibitor flavopiridol effectively prevented bone loss in two clinical relevant mouse models of acute and chronic induced bone loss. To our knowledge, this is the first detail report showing that flavopiridol treatment may be an effective treatment strategy for both clinical conditions of wear particle-induced osteolysis, and estrogenic-deficiency induced osteoporosis.

Under pathological conditions, the process of osteoclastogenesis is initiated by various innate and acquired mediators, including pro-inflammatory cytokines, which act on osteoclast precursors directly or indirectly, through the RANK/RANKL axis (Boyce and Xing, 2008; Xing et al., 2012). A previous study has shown that even a small rise in the level of systemic inflammation can precipitate osteodestruction (Sucur et al., 2014). Thus, investigation of factors that promote osteoclast activity may provide insight into the pathological events responsible for bone loss. In cases of acute bone osteolysis (Yang et al., 2016), joint injury (Yik et al., 2014; Hu et al., 2016), intervertebral disk degeneration (Wuertz et al., 2012) and osteoporosis (Adler et al., 2016), an exogenous stimulation triggers the secretion of various cytokines. Secreted cytokines include TNF-α, interleukin-1, interleukin-6, prostaglandin E2, and matrix metalloproteinases. These cytokines and factors induce inflammation and subsequently induce RANKL production by osteocytes, followed by recruitment of osteoclast precursors to the bone surface to initiate bone resorption.

Current anti-resorptive drugs available include bisphosphonates (e.g., Alendronate, Risedronate), calcitonin, selective estrogen receptor modulators (SEMRs), RANKL antibody (Denosumab) and Cathepsin-K inhibitor (Odanacatib) (Adler et al., 2016; Pazianas and Abrahamsen, 2016). However, side effects such as fever, throat and stomach ulcers, breast cancer, thromboembolism, hypercalcemia and hypertention, and also the bioavailability limit the use of these drugs in systemic treatment (Tian et al., 2014). Furthermore, despite these anti-resorptive drugs’ ability to reduce the risk of vertebral fractures by up to 70%, and hip fractures by 40–50%, there is only a 20–25% risk reduction for all other non-vertebral fractures (Díez-Pérez et al., 2014). Therefore, there is clinical demand for new therapeutic strategies, such as utilizing the bone protective effect of flavopiridol treatment to reduce fracture risk.

The interaction with RANKL induces trimerization of cell surface receptor RANK on osteoclast precursors (Xing, 2009; Xing et al., 2012), and subsequently activates TNF receptor associated factor 6 (TRAF6). This activates a cascade of intracellular events, such as the activation of NF-κB, mitogen-activated protein kinases (MAPKs), and the nuclear factor of activated T-cells 1 (NFATc1) signaling pathways, all of which are essential for osteoclast formation (Boyce et al., 2015). The classical NF-κB activation involves phosphorylation and degradation of IκBα, which then allows phosphorylation of NF-κB protein (such as p65) and its nuclear translocation to bind to DNA target sites to activate the expression of osteoclastogenesis-related genes (Abu-Amer, 2013). Genetic studies have confirmed that p65-knockout results in osteopetrosis due to the lack of osteoclast formation, demonstrating the essential role of NF-κB signaling in osteoclast differentiation (Boyce and Xing, 2008). Importantly, CDK9 interacts with p65 in the nucleus to modulate HIV-1 gene (Amini et al., 2002) and pro-inflammatory cytokine genes transcription (Brasier, 2008). Collectively, these studies suggest an inter-relationship between CDK9, NF-κB, and RANKL signaling pathway to regulate osteoclast differentiation. In this study, we confirmed that RANKL stimulation markedly activated NF-κB signaling pathway by phosphorylating p65 in BMM cells; however, flavopiridol treatment effectively suppressed p65 phosphorylation and nuclear translocation (**Figure 6**). As a result, osteoclastogenesis (**Figure 3**), osteoclast-specific gene expression (**Figure 4**), and osteoclast functions (**Figure 5**) were all suppressed in the presence of CDK9 inhibitors. Importantly, the above observation was made using non-cytotoxic levels of flavopiridol. Thus, our results provide strong evidence that the bone protective effects of flavopiridol is due to its inhibition of CDK9 activity (but not other CDKs,), which in turn is required for activation of RANKL and NF-κB signaling for osteoclastogenesis. In support of this, our previous result using CDK9 siRNA demonstrated that the anti-inflammatory activity of flavopiridol is due to specific inhibition of CDK9, but not other CDKs that regulate cell-cycle (Yik et al., 2014), which was also proved by another group (Oqani et al., 2011).

Various downstream effector genes are involved in the process of NF-κB (Boyce et al., 2010) activation, and among those genes, c-Fos and NFATc1 are the most important (Boyce et al., 2015). c-Fos is a major component of the transcription factor AP-1, which induces NFATc1, a master regulator of osteoclastogenesis, to regulate osteoclast formation (Iezaki et al., 2016). This explains our observation that inhibition of NF-κB by CDK9 inhibitors resulted in suppression of RANKL-induced c-Fos, and NFATc1, as well as the suppression of downstream osteoclast-specific markers such as TRAP, CTR, DC-Stamp, and CTSK.

There are potential limitations in this study. First, CDK9 also acts as a crucial transcription factor for other inducible genes. Therefore, we cannot exclude the possibility that besides NK-κB and RANKL, other unknown mediators and signaling pathways are also involved in flavopiridol-mediated repression of osteoclastogenesis. In addition, our study is limited to bone remodeling involving the calvaria and femur. Other skeletal system, such as the vertebral region, will need to be examined to broaden the implication and potential clinical applications.

In summary, our study conclusively demonstrates that flavopiridol treatment prevents bone loss in both acute and chronic induced bone loss mouse models. Flavopiridol treatment effectively suppresses osteoclastogenesis, osteoclast-specific gene expression, and osteoclast function *in vitro*. The mechanism of flavopiridol’s bone protective effects is in part due to its suppression of NF-κB and RANKL signaling pathways. Our study demonstrates that CDK9 is a potential therapeutic target for disease modifying treatment of bone-related illness.

## Author Contributions

ZH and SF: study design. ZH, YC, and LS: study conduct. YC and LS: data collection. ZH and YC: data analysis. ZH, JY, and SF: data interpretation. ZH, JY, and DH: drafting manuscript. ZH, DH, and SF: revising manuscript content. SF: integrity of the data analysis. All authors approving final version of manuscript. We thank all authors for their contribution to this work.

## Conflict of Interest Statement

The authors declare that the research was conducted in the absence of any commercial or financial relationships that could be construed as a potential conflict of interest.
