# Supplementary material for: Flavopiridol Protects Bone Tissue by Attenuating RANKL Induced Osteoclast Formation
Source: Front Pharmacol. 2018 May 3;9:174. doi: 10.3389/fphar.2018.00174 (PMC5944179; doi:10.3389/fphar.2018.00174)
Supplement: Supplementary file 1 [file Image_1.PDF]

Supplementary Material

Flavopiridol protects bone tissue by attenuating RANKL induced osteoclast formation

Zi'ang Hu, M.D., Ph.D [1], Yilei Chen, M.D. [1], Lijiang Song, M.D. [1], Jasper H. N. Yik, Ph.D [2], Dominik R. Haudenschild, Ph.D [2], Shunwu Fan, M.D. [1,*]

*** Correspondence:**

Shunwu Fan,

Department of Orthopaedic Surgery, Sir Run Run Shaw Hospital, Medical College of Zhejiang University, 3 East Qingchun Road, Hangzhou 310016, China.

Tel.: 0086-13505819572;

Fax: 0086-571-86044817

Email: 11118149@zju.edu.cn

Supplementary Figures

Supplementary Material 1

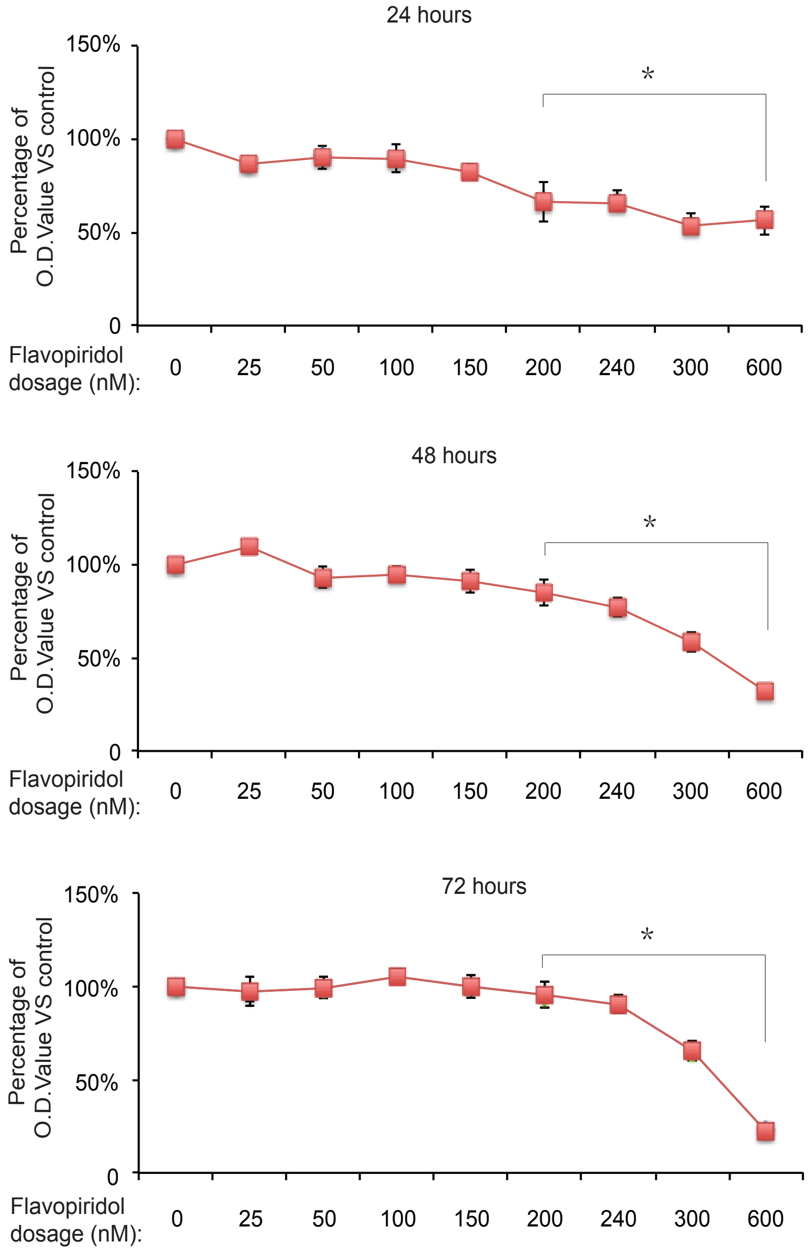

Supplementary Figure 1.

Maximum *in vitro* non-cytotoxic concentration (200 uM) of flavopiridol treatment in BMMs. Percent viability of flavopiridol-treated BMMs after 24 (A), 48 (B), and 72 hours (C), compared to untreated controls. Results were the mean +/- standard deviation (n=3, **P*< 0.05, One-way ANOVA). The maximum flavopiridol dose that was tolerated by cells without a significant drop in viability was determined to be 200 nM.
